# Antibiotics: from use to abuse

**DOI:** 10.1590/S1808-86942012000200001

**Published:** 2015-10-20

**Authors:** Luc Weckx

**Affiliations:** Full Professor of ENT – Federal University of São Paulo School of Medicine – UNIFESP – Escola Paulista de Medicina. Treasurer of the Brazilian Medical Association. Board Member of the Paulista Association for the Development of Medicine (SPDM). Vice-Chairman of COLSAN

In 1928, the discovery of penicillinand its production for large scale use in the early 40's, had a major impact on the Second World War; my family witnessed this fact when my father had appendicitis, which complicated with peritonitis, without much hope for survival, was saved by the administration of penicillin, acquired through much difficulty at a military hospital. Today, we live the opposite situation, a true abuse concerning the use of antibiotics. Even without knowing about it, every day we “feed ourselves antibiotics”, which are included in the feed of cattle and in agriculture. Another abuse factor is the inadequate use of antibiotics. For instance, 50%to 60% of the patients with colds or flues –which are caused by viruses and, therefore do not respond to antibiotics, make unnecessary use of antibiotics.

This increase in the use of antibiotics in the community eliminates the weaker bacteria and selects the stronger ones, in other words, it breeds superbacteria, resistant to multiple antibiotics, responsible for the hospital infections which today kill more Americans than AIDS. It has been proven that the increase in bacterial resistance grows in parallel with the increase in antibiotic consumption by the community. On the other hand, while bacteria are champions in evolution – having been around for 4 billion years, research for new antibiotics has been on the downfall since 1980, because the pharmaceutical industry is prioritizing the search for more lucrative drugs, such as those of prolonged use for diabetes and cancer. It suffices to remember that the amoxicillin-clavulanic acid association, indicated for bacterial infections of the upper airway (H.influenza andmoraxella)resistant to simple amoxicillin, is “celebrating” 30 years of existence this year.

If we consider that Upper Airway Infections (UAW) infections and pneumonia are responsible for 60% of outpatient prescription of antibiotics, and the most common UAWI, the cold, may happen 2 to 4 times a year in the adult and as often as 6 to 12 times per year in children, we understand that our role as otorhinolaryngologists, must involve an attempt to solve this problem. In 2011's World Health Day, the World Health Organization (WHO) published a warning about the risk of bacterial resistance associated with the “injudicious use of antibiotics”. Even worse than the bad indication, infectologists Carlos Kiffer and Antonio Carlos Pignatari, from UNIFESP, recently published a study based on the prescriptions issued by 400 physicians in São Paulo, suggesting that the doses of the antibiotics prescribed for pneumonia, pharyngitis and sinusitis were lower than the correct ones indicated to eradicate those respiratory pathogens.

Today, because of Resolution RDC 20/2011 from Anvisa – which obligates Brazilian pharmacies to only sell antibiotics for patients who have a medical prescription (in two copies), we physicians can no longer blame the patients' grandparents and pharmacy attendants for self-medication and incorrect use of antibiotics. We need to be updated concerning the new scientific evidence which tries to preserve the efficacy of the existing antibiotics available in our country, following the Guidelines of the Brazilian Medical Association and ABORL Consensus; such as, for example not to recommend routine antibiotics in uncomplicated sinusitis, tonsillitis or acute otitis media. Moreover, we must be aware of new vaccines, such as the flue and the pneumococcus vaccines, which, by reducing the number of acute infections of the airways, they reduce the need to use antibiotics and, therefore, help reduce bacterial resistance. We also hope, for a near future, to have viral and bacterial biomarkers, such as Procalcitonin (elevated in bacterial processes), viral and bacterial PCR, in order to increase the bacterial diagnosis accuracy, to know, for instance: “is it still a flue, or has it complicated and is now a purulent acute sinusitis?”

Parallel to this, international epidemiological surveillance studies are trying to identify emergent superbacteria in different regions of the planet; and there are simple measures, valid for all health care professional and for the population in general, such as washing one's hands when in contact with infected patients, to help prevent the transmission of resistant bacterial strains, which are also fundamental in order to try to contain what the WHO considers to be one of the greatest global health challenges: the increase bacterial resistance towards antibiotics.

